# The Correlation Between Mechanical Ventilation Duration, Pediatric Sequential Organ Failure Assessment Score, and Blood Lactate Level in Children in Pediatric Intensive Care

**DOI:** 10.3389/fped.2022.767690

**Published:** 2022-03-14

**Authors:** Fang Lu, Hua Qin, Ai-Min Li

**Affiliations:** ^1^The Second Clinical Medical College, Yangtze University, Jingzhou, China; ^2^Department of Pediatrics, The Second People's Hospital of Jingmen, Jingmen, China

**Keywords:** duration of mechanical ventilation, pSOFA score, lactate, PICU, operator characteristic

## Abstract

**Objective:**

This study aimed to investigate whether the ventilation duration for children undergoing invasive mechanical ventilation (IMV) in pediatric intensive care unit (PICU) is correlated with pediatric sequential organ failure (pSOFA) score, white blood cell (WBC) count, blood lactate level, and duration of fever.

**Methods:**

Retrospectively reviewed that the medical records of patients who received IMV in the PICU of Jingzhou Central Hospital between January 2018 and December 2020. According to the duration of IMV in diagnosis-related groups, these patients were divided into two groups: group A, ventilation duration <96 h, and group B, ventilation duration ≥96 h. Each group's pSOFA scores, WBC counts, blood lactate levels, and durations of fever were compared. Logistic regression analysis was used to analyze the clinical risk factors of ventilation duration ≥96 h, and the receiver operator characteristic (ROC) curve was drawn.

**Results:**

A total of 42 patients were included, including 23 in group A and 19 in group B. The difference in pSOFA score between group A and group B was statistically significant (*P* < 0.05), while the differences in blood lactate level, duration of fever, and WBC count between the two groups were not statistically significant (*P* > 0.05). Logistic regression analysis was conducted to analyze the influencing factors of mechanical ventilation duration ≥96 h. An ROC curve was drawn with pSOFA score as a test variable and duration of mechanical ventilation ≥96 h as a state variable, revealing that the area under the curve was 0.76 (SE = 0.075, 95% CI: 0.614–0.906, *P* = 0.005). The sensitivity and specificity were 68.4 and 73.9%, respectively, and the corresponding pSOFA score was 7.5.

**Conclusion:**

When the pSOFA score ≥8, the risk of mechanical ventilation duration ≥96 h increases.

## Introduction

In PICU, ventilation is widely used and recognized as an important auxiliary treatment method. The duration of mechanical ventilation refers to the time patients receive invasive respiratory support through tracheal intubation. However, ventilator use also increases the risk of complications, such as ventilator-associated pneumonia (VAP), right ventricular dysfunction, and respiratory muscle injury (1), prolonging a patient's hospital stay and increasing both medical costs (2) and mortality rates. VAP is the second most common hospital-acquired infection resulting from PICU hospitalization (3). Previous research has found that the longer the duration of mechanical ventilation, the higher the risk of VAP (4). A previous study identified mechanical ventilation duration >96 h as a risk factor of VAP (5), and ventilator-related events have been found to be common in ICU patients with mechanical ventilator duration ≥96 h (6).

With the 2016 release of Sepsis 3.0, which is based on SOFA scores, there was a lack of a scoring system for children. An age-adaptive sequential failure scoring system for children has since been developed based on the SOFA score, in which the scoring of the liver and kidney function, circulatory system, and nervous system of children of different ages were standardized to provide criteria for the diagnosis and severity of sepsis in children. The aim of this system is to address the deficiency in children's diagnosis (7). As of 2018, there are three pSOFA scoring systems: one proposed by Schlapbach et al. (8), one proposed by Matics et al. (9), and one proposed by Shime et al. (10). The first two systems have been verified (11), and the second is widely used in clinical practice, leading to more accurate prediction of mortality rates (12–14).

In the present study, the clinical data of 42 children who received IMV in the PICU of Jingzhou Central Hospital between January 2018 and December 2020, were collected and retrospectively analyzed to identify differences between pSOFA score, WBC count, blood lactate level (within 6 h before intubation), and duration of fever. The objective was to investigate whether the duration of IMV for children in PICUs is correlated with pSOFA score.

## Information and Methods

### Subjects

Eligible children with mechanical ventilation admitted to the PICU of Jingzhou Central Hospital from January 2018 to December 2020 were selected. Inclusion criteria: (1) clear diagnosis of primary disease; (2) duration of mechanical ventilation ≥24 h; (3) patients with complete data. Exclusion criteria: (1) age < 31 days or > 12 years; (2) patients with surgical disease or surgical operation; (3) patients with midway transfer, abandonment of treatment, or death; (4) patients with incomplete case data.

A total of 42 patients were included, including 23 in group A and 19 in group B, aged between 1 and 74 months. The median age of group A was 2 months, that of group B was 3 months, and the difference was statistically significant (*P* > 0.05). Of these children, 39 had severe pneumonia, 1 had myocarditis, 1 had undergone bronchial foreign-body removal, and 1 had diabetic ketoacidosis. Forty of the children had been given sedatives and analgesic drugs (see Table 1).

**Table 1:** Baseline demographics of two groups.

	**Number, %**		***P***
	**Group A**	**Group B**	
**Age**	
Median (number)	2 months (23)	3 months (19)	0.823
**Gender**
Male	14 (60.9%)	10 (52.6%)	0.591
Female	9 (39.1%)	9 (47.4%)	
**Diagnosis**	
Severe pneumonia	20 (86.8%)	19 (100%)
Myocarditis	1 (4.3%)
Foreign body inhalation	1 (4.3%)
Diabetic ketoacidosis	1 (4.3%)	

*Group A: duration of mechanical ventilation <96 h, Group B: duration ≥96 h*.

### Observation Indices

The medical data collected included gender, age, IMV duration, blood lactate level (within 6 h before intubation), duration of fever (from admission to transferring out of PICU), Duration of stay, total bilirubin (within 24 h before intubation), creatinine (within 24 h before intubation), platelet count (within 24 h before intubation), PiO_2_/FiO_2_ value or SpO_2_/FiO_2_ value, Glasgow coma scale score, blood pressure, use of circulatory support drugs, WBC count (within 24 h before intubation), hospital stay and pSOFA score at the beginning of IMV. According to the duration of mechanical ventilation in diagnosis-related groups, the patients were divided into two groups: group A, ventilation duration <96 h, and group B, ventilation duration ≥96 h.

### Statistical Analysis

Statistical analysis was conducted using statistical software SPSS 23.0. Normally distributed measurement data were expressed as mean ± standard deviation (*x* ± SD) and compared using an independent sample *t*-test, while non-normally distributed measurement data were expressed as the median and interquartile and compared using a non-parametric Mann–Whitney *U* test. Logistic regression analysis was conducted to analyze the influencing factors of mechanical ventilation duration ≥96 h. The receiver operator characteristic (ROC) curve method was used to analyze the best cut-off value, sensitivity, and specificity of a predictive exponential equation in predicting pSOFA scores in the case of IMV duration ≥96 h. *P* < 0.05 was considered statistically significant.

## Results

### Comparison of Clinical Indexes Between the Two Groups

The pSOFA score was significantly higher in group B than in group A (*p* < 0.05, Table 2). The differences in blood lactate level, duration of fever, hospital stay, and WBC count between the two groups were not statistically significant (*P* > 0.05).

**Table 2:** Comparison of pSOFA score, lactate level, duration of fever, hospital stay and WBC count between two groups.

**Factor**	**Group A (*n* = 23)**	**Group B (*n* = 19)**	**t/Z**	***P***
pSOFA score	6.5 ± 2.0	8.0 ± 1.2	−2.775	0.008
WBC (10^∧^9/L)	7.05 (5.33~9.99)	7.2 (5.4~12.48)	−0.746	0.436
Lactate (mmol/L)	2.0 (1.3~3.1)	1.3 (0.8~2.7)	−1.468	0.257
Duration of fever (day)	4 (3~6)	5 (3~10)	−0.918	0.358
Hospital stay (day)	13.2 ± 4.3	18 ± 5.6	−3.146	0.003

*Group A: duration of mechanical ventilation <96 h, Group B: duration ≥96 h*.

### Univariate Analysis of Duration of Mechanical Ventilation ≥96 Hours

The related factors with statistical significance between the two groups were used for logistic regression analysis in order to identify the influencing factors of mechanical ventilation duration ≥96 h. The results of the analysis revealed that pSOFA score (OR = 1.694, 95% CI: 1.099–2.610) is an independent risk factor.

**Figure 1:**
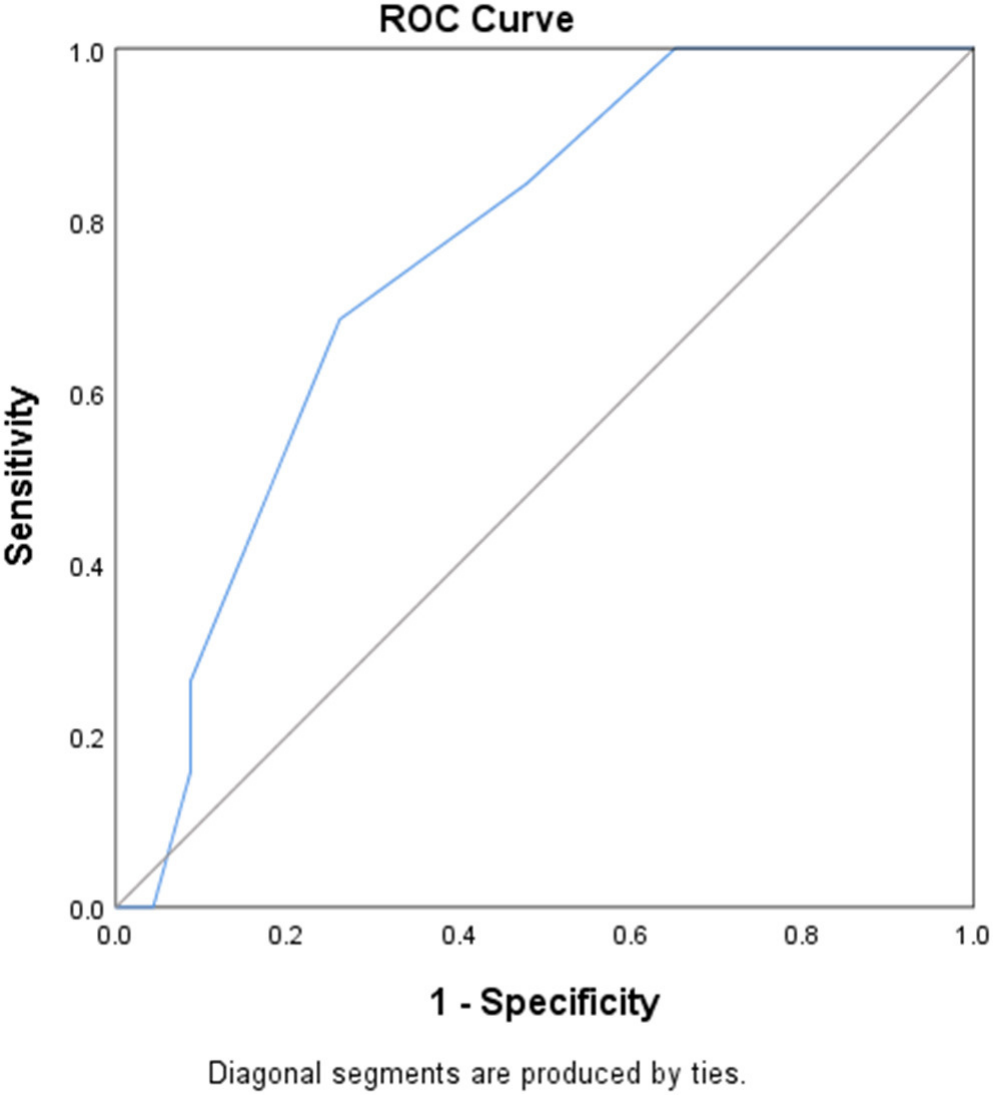
ROC curve of the risk factors. Diagonal segments are produced by ties.

An ROC curve was drawn with pSOFA score as a test variable and duration of mechanical ventilation ≥96 h as a state variable. This revealed that the area under the curve was 0.76 (SE = 0.075, 95% CI: 0.614–0.906, *P* = 0.005), the sensitivity and specificity were 68.4 and 73.9%, respectively, the Youden's index was 0.423, and the corresponding pSOFA score was 7.5; that is, when pSOFA score ≥ 8, the risk of duration of mechanical ventilation ≥96 h is relatively higher (see Figure 1).

### The Mean Duration of Hospital Stay in Children With SOFA Score of More Than or Less Than 8

The mean duration of hospital stay in children with SOFA score of more than 8 was 16.6 ± 5.6 days, that of <8 was 14.4 ± 5.2 days, the different was not statistically significant (*P* > 0.05, see Table 3).

**Table 3:** The mean duration of hospital stay in children with SOFA score of more than or less than 8.

**Factor**	**pSOFA score <8** **(*n* = 23)**	**pSOFA score ≥8** **(*n* = 19)**	**t**	***P***
Hospital stay (day)	14.4 ± 5.2	16.6 ± 5.6	−1.274	0.210

## Discussion

The pSOFA score predicts the severity and prognosis of sepsis in children more accurately than pediatric risk score of mortality and pediatric critical illness score (12–15) and can also be used to predict the outcome of the general PICU population. Furthermore, it is more accurate than systemic inflammatory response syndrome (SIRS) in defining pediatric sepsis (16) and can better predict the new incidence rate or mortality rate of critically ill children within 3 years (17). Matics et al. found that the best threshold of pSOFA for differentiating mortality rate was >8 (9). In another single center study, a pSOFA Score >4 was associated with a significant increase in mortality (18).

In PICU, the use of ventilators is closely related to the occurrence of VAP and other complications. Prolonged mechanical ventilation has been found to increase the use of broad-spectrum antibiotics (19), lengthen the hospitalization duration for children due to longer recovery, and increase the risk of death in children with VAP (20).

In the present study, the best critical value of pSOFA for differentiating ventilation duration ≥96 h was eight points. This is also confirmed to a certain extent by the findings of Matics et al. and Lalitha et al. found that when pSOFA score was more than 8 on both days 1 and 3, the relative risk of prolonged duration of mechanical ventilation was increased (21). When the initial pSOFA score at the beginning of ventilator use is ≥8, the risk of mechanical ventilation duration ≥96 h is higher, so taking effective preventive measures in nursing and treatment can improve patient outcomes (22). For children with a score >8, chest radiographs should be performed 72 h after mechanical ventilation, so that VAP can be detected and treated early.

Blood lactate level is an indicator of hypoxia, cancer (23), and the body's response to an epinephrine infusion can also cause the lactate level to rise (24). Lactate clearance rate has certain significance to evaluate the prognosis and curative effect of critically ill patients, and a decrease in lactate clearance rate can cause a decrease in oxygen metabolism ability, leading to hypoxia and damage in the tissues and organs. Choi et al. found that blood lactate level and mechanical ventilation were significantly correlated with mortality rate (25), and a recent study revealed that 24-h lactate level was an independent predictor of mortality rate, which was positively correlated with pSOFA, indicating that 24-h lactate level into pSOFA score can lead to more accurate prediction (26). Six-hour lactate level has also been found to be better than 0-h lactate level for predicting mortality (13). In the present study, only 0-h lactate level was measured, and it was found that there was no correlation between 0-hlactate level and duration of mechanical ventilation. One study found that 6-h lactate level was associated with mortality and superior to 0-h lactate levels (13). This study did not measure 6-h lactate level and lactate clearance rate, however, so whether 6-h lactate level and lactate clearance rate are related to the duration of mechanical ventilation requires further study.

The present study was a retrospective study and had three limitations: (1) there may have been recall bias and selection bias; (2) the incidence of VAP was not analyzed; (3) the significance of blood lactate level in the judgment of duration of mechanical ventilation in critically ill children is still not clear, for which further research is required.

## Conclusion

Logistic regression analysis was used to establish the ROC curve and predict the sensitivity and specificity of pSOFA scores at the beginning of mechanical ventilation, which is of great significance to the clinical development of individualized and standardized preventive treatment measures to reduce the risk of VAP and other mechanical ventilation complications. It was found that, when the pSOFA score ≥ 8, the risk of mechanical ventilation duration ≥96 h increased. Taking active preventive measures early may minimize the incidence of ventilator-associated pneumonia and other complications, shorten the length of the hospital stay for the patient, and reduce costs.

## Data Availability Statement

The original contributions presented in the study are included in the article/supplementary material, further inquiries can be directed to the corresponding author.

## Ethics Statement

The study was conducted in accordance with the Declaration of Helsinki (as was revised in 2013). The study was approved by Ethics Committee of Jingzhou Center Hospital-the Second Clinical Medical College of Yangtze University.

## Author Contributions

FL and HQ: conception and design of the research. HQ: acquisition of data. FL: analysis and interpretation of the data. A-ML: statistical analysis, obtaining financing, and critical revision of the manuscript for intellectual content. FL and A-ML: writing of the manuscript. All authors read and approved the final draft.

## Funding

This study was funded by the Jingzhou Science and Technology Bureau (No. 2019CC54-03).

## Conflict of Interest

The authors declare that the research was conducted in the absence of any commercial or financial relationships that could be construed as a potential conflict of interest.

## Publisher's Note

All claims expressed in this article are solely those of the authors and do not necessarily represent those of their affiliated organizations, or those of the publisher, the editors and the reviewers. Any product that may be evaluated in this article, or claim that may be made by its manufacturer, is not guaranteed or endorsed by the publisher.
